# Country-specific determinants of world university rankings

**DOI:** 10.1007/s11192-017-2634-1

**Published:** 2017-12-23

**Authors:** Jacek Pietrucha

**Affiliations:** University of Economics, Katowice, Poland

**Keywords:** Ranking indicators, Universities, Political stability, Research policy, Educational system

## Abstract

This paper examines country-specific factors that affect the three most influential world university rankings (the Academic Ranking of World Universities, the QS World University Ranking, and the Times Higher Education World University Ranking). We run a cross sectional regression that covers 42–71 countries (depending on the ranking and data availability). We show that the position of universities from a country in the ranking is determined by the following country-specific variables: economic potential of the country, research and development expenditure, long-term political stability (freedom from war, occupation, coups and major changes in the political system), and institutional variables, including government effectiveness.

## Introduction

World university rankings (WUR) have become very popular in recent years. They follow different methodologies to assess the relative impact of each university on science and teaching (Rauhvargers 2011). At the same time, these rankings are sometimes overestimated in public debate as a mirror reflection of the efficiency of research and the higher education system. Consequently, rankings are used in public debate to discuss the quality of university management and the necessity for reform in research and higher education (Hazelkorn 2007; AUBR 2010; Saisana et al. 2011).

There are a number of studies that explore the factors that determine the positions of universities in international rankings. A few papers have researched determinants such as academic quality management (McCormack et al. 2014). Aghion et al. (2010) and McCormack et al. (2014) have pointed out that more autonomous universities that need to compete more for resources are more productive. Marconi and Ritze (2015) show a relationship between a university’s score in international university rankings and its expenditure per student and other factors, such as the university mission, size and productive inefficiency. In an extensive number of studies the shortcomings of the rankings have been pointed out due to their size, language, and hard science bias (e.g. Taylor and Braddock 2007; Saisana et al. 2011; Moed 2017). Safon (2013) has looked for hidden factors, such as the reputation of a country, that are not related to the quality of modern universities and are determinants of the influential rankings.

This paper aims to investigate more deeper determinants of the position of universities in the rankings. The study is based on a basic intuition that there are common factors that determine the international position of universities from a given country. These country-specific factors comprise not only economic variables (such as GDP), but also the longevity and stability of the state, and the quality of institutions. This paper is structured as follows: first the methodology of the three leading rankings and their shortcomings is briefly described; secondly the methodology and data used in this study are outlined, and finally the results are discussed.

## University rankings

The number of university rankings (national and international) is growing every year. Some of the most prominent world university rankings include: the Academic Ranking of World Universities (ARWU), the QS World University Ranking, and the Times Higher Education World University Ranking.

The Academic Ranking of World Universities (ARWU), also known as the Shanghai Ranking, is conducted by researchers at the Centre for World-Class Universities at Shanghai Jiao Tong University, and published by Shanghai Ranking Consultancy. A description of the ranking can be found in Liu and Cheng (2005). The ARWU is based on four criteria: (1) quality of education, (2) quality of faculty, (3) research output, and (4) academic performance. Universities that have Nobel laureates, Fields medalists, Highly Cited Researchers, or papers published in *Nature* or *Science* are included in the ranking. In addition, universities with a significant number of papers indexed by the Science Citation Index-Expanded (SCIE) and Social Science Citation Index (SSCI) are also included (ARWU 2016).

The QS World University Ranking is based on six performance indicators designed to assess universities from four perspectives: research, teaching, employability and internationalization by collecting information from both public databases and global surveys of academics and graduate employers. Performance indicators comprise: academic reputation, employer reputation, student-to-faculty ratio, citations per faculty, international faculty ratio, and international student ratio. Academic reputation is measured using a global survey, in which academics are asked to identify the institutions where they believe the best work is currently taking place within their own field of expertise. In the same vein the employer reputation indicator is also based on a global survey, asking employers to identify the universities they perceive to be producing the best graduates (QS 2016).

The Times Higher Education World University Ranking employs 13 performance indicators that are grouped into five areas: teaching (reputation survey, staff-to-student ratio, doctorate-to-bachelor’s ratio, doctorates-awarded to-academic-staff ratio, and institutional income), research (reputation survey, research income, research productivity), citations (research influence), international outlook (staff, students and research), and industry income (knowledge transfer). The most prominent indicators look at a university’s reputation for research and teaching excellence among its peers, based on the responses to the annual Academic Reputation Survey (TE 2016).

The WUR are exposed to a great deal of criticism (Taylor and Braddock 2007; Billaut et al. 2010; Longden 2011; Moed 2017 among others). The main area of the critique addresses issues specific to creating composite indicators. There is always a question of whether the qualitative measure was suitably selected, as there is a lingering sense that some important information might have been missed, and the selection of weights is usually more or less subjective.

A study done by Soh (2017) summarized the methodical and statistical shortcomings of the WUR. Most criticism is directed at the simple weight-and-sum approach generally used in such rankings. Soh counted seven deadly sins of the WUR: spurious precision (rankings overestimate small differences in the total score), weight discrepancies, assumed mutual compensation, indicator redundancy, an inter-system discrepancy, negligence of indicator scores, and an inconsistency between changes in ranking and overall scores. These methodical weaknesses limit the reliability of the WUR. On the other hand, some of these shortcomings are common for all highly aggregated indicators. No choice of a method to create such indicators is free of criticism. It is possible to formulate similar objections to almost all highly aggregated indicators (e.g. GDP or CPI).

The most prominent other fields addressed by critics include natural science- size- language- and research bias. (1) The arts, humanities, and to a large extent social sciences are underrepresented in the rankings. This relative neglect stems from persistent biases that remain in bibliometric indicators (Rauhvargers 2011), which favour medicine, natural sciences and engineering. These constitute the most prominent fields in the Thomson Reuters and Elsevier databases, and therefore determine, to a large degree, performance in the global rankings (Rauhvargers 2013). (2) There is a bias towards size dependency, especially in the ARWU (Waltman et al. 2012; Docampo and Cram 2015). (3) Universities in the U.S. and in other English-speaking countries dominate the global rankings. Research written in languages other than English is read and cited less (van Raan et al. 2011; Rauhvargers 2011). (4) Global university rankings reflect university research performance far more accurately than teaching (Rauhvargers 2011).

Hidden factors or profiles are also investigated to explore the criteria adopted in the rankings. Safon (2013) reviews underlying factors in university rankings such as: language, country, size (of university), age (of university), scope and research focus, and the reputation factor.

Bearing all the above shortcomings and reservations in mind, there are two reasons for the investigation of factors determining the position of a given country in the rankings. Firstly, these measurements, although far from perfect, are the only ones available. More importantly, however, despite their many shortcomings, biases and flaws, the rankings enjoy a high level of acceptance among stakeholders and the wider public, resonate powerfully with the media, and serve as an argument in public debates and private decisions about choosing a university.

In connection with the methodological doubts concerning the WUR for robustness check, it is worth performing an analogous study using a ranking created according to another methodology. Our research was extended using the Ranking of National Higher Education Systems published by Universitas 21. The ranking includes 25 measures of performance grouped into four modules: Resources, Environment, Connectivity, and Output, and intentionally includes countries as the unit of analysis (U21 Ranking 2016), according to the methodology described in: Williams et al. (2013). In many ways this indicator takes a different approach, and was created including the criticism which is formulated towards WUR.

## Methods and data

World university rankings are focused on the individual performance of each university. Accordingly, while investigating the international position of a university, determinants at the micro level such as academic quality management may be taken into account. This research assumes a different perspective. We are looking for country-specific factors that determine the international position of universities from that country. The focus of the study is on determinants such as characteristics of the state and its economy. Speaking in the language of economics, the macro perspective has been adopted. This derives from a belief that all universities from a given country share common characteristics that determine their position in the rankings. Some of the determinants have been examined in previous research (e.g. language). Some others, to the best of our knowledge, have not been investigated in a systematic way. The latter include the longevity of the state, its stability, and freedom from armed conflict and internal political upheavals.

In order to investigate the factors determining the position of universities from a given country in university rankings, a cross-sectional regression was run. The parameters were estimated using OLS. The dataset used in the paper covers 42–71 countries (depending on the ranking and data availability). All the models were estimated in two versions: for different number of the countries (our baseline estimates) and for the same *N*. In the latter case, only the countries that appear in all the rankings were included. A negative consequence is the considerable decrease in *N*. We have assumed that we cannot obtain identical Ns in a way that a country not listed in the ranking (e.g. LS) is assigned with 0. The absence of a country in the ranking may be based on various grounds (ranking scope, data shortage, etc.) and not only on a low assessment of the universities. All baseline models (apart from U21 with 47 cases, where heteroscedasticity is found) passed standard tests, e.g. Shapiro–Wilk for testing normality, and Breusch–Pagan–Godfrey for testing heteroscedasticity. In the case of U21 we re-estimated the model using heteroscedasticity-consistent standard errors.

The essential problem in the presented empirical study was that a lack of existing research in this field made it impossible to rely on a well-verified group of control variables. We have selected a group of variables that from the perspective of a wider context appear to be substantially most reasonable, and then the we tested statistical significance using stepwise regression. For robustness check we have also estimated other models—but only the results for forward stepwise regression are presented.

For each of the rankings, an indicator was created to define the position of a country in the ranking while retaining the number of universities examined in the ranking and the presentation of the results. Each university was assigned with a value depending on its position in the ranking. The specificity of each ranking was maintained. Then, the values for universities of a given country were summed up. For example, the ARWU indicator was calculated in the following way: a university that came first in the ranking was granted 500 points, while universities that came between 401 and 500 were granted 100 points. The position of a country in the ranking is determined by the sum of the values obtained for each of the universities. An analogous indicator was calculated for the Times Higher Education World University Ranking: the leading position in the ranking was granted 700 points, whereas position 601+ was granted 100 points. As the distribution of the raw data is asymmetric, we use log values.

The following independent variables were examined.

### The size of the economy

Which is intuitively based on the fact that the value of production and income in the economy impacts the capacity to sustain academic excellence. The size of the economy was measured as a log of GDP.

### The level of economic development

Which impacts funding capacity (both in the private and public sectors). The level of economic development was measured as a log of GDP per capita.

### Long-term political stability and the longevity of the state structure

Wars, occupations, revolutions, and coups have a disturbing effect on the continuity of universities, and they often reduce or even annihilate their potential. Considering the long-term and partially cumulative nature of knowledge and skills (also in the narrow context of university management), such events have a long-term effect and are often irreversible, even over extended periods of time. A number of countries in Central and Eastern Europe are a case in point. From 1939 to 1945, Poland, under German and Soviet occupation experienced acts of grim repression targeted against intellectuals. The shifting borders after 1945 meant that major academic centres were either closed down or relocated. Stalinist and communist repression in the 1950s dealt another blow to the intellectual potential of Poland. In the years that followed, the policy of the communist regime produced waves of political migration (especially in 1968 and the 1980s). Unfortunately, none of the existing indicators are able to measure this kind of instability (i.e. the indicator value should grow as the period of freedom from instability continues). For this reason, a new indicator was created based on the Polity IV research project.

The Polity IV Project provides an assessment of autocratic and democratic traits inherent in a political system. The Polity score is computed by subtracting the Autocracy score from the Democracy score. At the same time, the assessment takes into account periods of instability in the system, wars, anarchy, etc. As a consequence, the result is a unified polity scale that ranges from + 10 (strongly democratic) to − 10 (strongly autocratic), with special values (66, 77, 88) for cases of foreign “interruption”, cases of “interregnum” or anarchy, etc. (Marshall et al. 2016). Accordingly, an indicator was created that represented the number of years from the last major political instability until 2015. The following interpretation of the indicator was provided: a growth in value reflects the growing number of years that have elapsed since the last major period of political instability.

This indicator is similar to the Durable indicator from Polity IV, but ours covers only major changes in the political system (10 points and more). In other words, the new indicator measures freedom from war, occupation, coups, and major changes in a political system; it fails to assess political instability involving frequent changes of government or minor adjustments to the political system.

### R&D expenditure

According to a number of studies, the funding of research (both from public and private sources) is a key determinant of its success. World Bank data on R&D expenditure were used.

### Quality of institutions

Universities are part of the institutional and governance arrangements of the country. Institutional arrangements affect the universities in direct (via, e.g. legislation related to the educational system of the country or knowledge transfer) and indirect ways (via general public policy and regulatory quality, etc.). World Bank data from The Worldwide Governance Indicators (WGI) project were used (World Bank 2017). Governance consists of the traditions and institutions by which authority in a country is exercised. Two indicators were considered in the study: Regulatory quality and Government effectiveness. Government effectiveness captures perceptions of the quality of public services, the quality of the civil service and the degree of its independence from political pressures, the quality of policy formulation and implementation, and the credibility of the government’s commitment to such policies. Regulatory quality captures perceptions of the ability of the government to formulate and implement sound policies and regulations that permit and promote private sector development (World Bank 2017). As they are significantly correlated with each other, the results can finally be interpreted more broadly from the point of view of the quality of institutions and public policy.

Detailed information on variables and sources of data is provided in Table 1. Due to specific rules governing the rankings, data on the position of each country in the rankings were obtained from rankings published for the year 2016, while the values of dependent data come from 2015. In several cases the ultimate number of the countries captured in the research was smaller than in the ranking due to missing data on independent variables. The relatively largest gap in the data was found in R&D expenditure.

**Table 1:** Data sources

Abbreviated name	Variable	Source
ARWU	Academic Ranking of World Universities 2016	http://www.shanghairanking.com
THE	The Times Higher Education World University Ranking 2015–2016	http://www.timeshighereducation.com
QS	QS World University Rankings 2015–2016	http://www.topuniversities.com
U21	U21 Ranking of National Higher Education Systems, 2016	U21 (2016)
GDP	Gross domestic product based on purchasing power parity (PPP), 2015	World Bank, World Development Indicators
GDP pc	Gross domestic product per capita based on purchasing power parity (PPP), 2015	World Bank, World Development Indicators
STA	Long-term political stability—2017 number of the years without major changes in polity index, 2015	Polity IV; own calculation
REQ	Regulatory quality, 2015	World Bank, Worldwide Governance Indicators
GE	Government effectiveness, 2015	World Bank, Worldwide Governance Indicators
R&D	Research and development expenditure (% of GDP), 2015	World Bank, World Development Indicators
English	Dummy variable for English speaking countries	

## Results

The basic estimates from the cross-sectional regression are reported in Tables 2 and 3.

**Table 2:** Results for baseline regressions

	ARWU	THE	QS	U21
GDP	0.517*** (6.034)	0.557*** (7.964)	0.696*** (13.443)	
GDP pc	0.326*** (2.965)			0.239** (2.629)
R&D	0.236** (2.469)	0.335*** (4.077)		0.186***(3.014)
STA	0.297*** (3.094)		0.129** (2.143)	0.390*** (6.630)
REQ		0.311*** (3.505)		
GE			0.419*** (7.135)	0.300***(2.868)
ENG		0.182** (2.603)		
*N*	42	61	71	47
*R* ^2^	0.79	0.79	0.83	0.89
Adjusted *R* ^2^	0.76	0.78	0.82	0.89

Standardized coefficients are reported. In parentheses, *t*-statistics are reported. Significant coefficients are denoted with stars (*p < 0.1; **p < 0.05; ***p < 0.01)

**Table 3:** Results for same number of countries

	ARWU	THE	QS	U21
GDP	0.495*** (5.219)	0.676*** (6.882)	0.775*** (9.374)	
GDP pc	0.276** (2.243)			0.288*** (3.012)
R&D	0.254** (2.459)			0.166** (2.544)
STA	0.358*** (3.361)	0.253** (2.230)	0.266*** (2.786)	0.379*** (5.936)
REQ		0.481*** (4.124)	0.377*** (3.839)	
GE				0.307*** (2.834)
ENG				
*N*	39	39	39	39
*R* ^2^	0.76	0.76	0.82	0.91
Adjusted *R* ^2^	0.73	0.74	0.81	0.90

Standardized coefficients are reported. In parentheses, *t*-statistics are reported. Significant coefficients are denoted with stars (*p < 0.1; **p < 0.05; ***p < 0.01)

One of the key factors determining the standing of a university in the WUR is the size of the country’s economy. One possible interpretation is that GDP reflects the economic potential of a country, which easily translates into the funding necessary for securing academic excellence at universities. The variable directly measuring the funding stream (broadly defined as R&D) is also in most cases (apart from QS) statistically significant. This confirms the pivotal (and well-known) role of funding in science (from various sources). U21 takes into account financing of universities in the Resources section (different measures of expenditure on tertiary education institutions). Examination of the relationships between subindexes confirms, however, the importance of funding for the research output and the quality of a nation’s best universities (U21 Ranking 2016).

Most of the results demonstrate explicitly that the level of economic development has little effect on WUR. In other words, taking other variables into account, GDP per capita is not relevant to world university rankings (apart from ARWU). Considering the adopted methodology, the result remains robust when faced with any change in specifications. This result is attributed to the fact that the research captured a large number of countries with a relatively low level of GDP per capita (e.g. India, China, Brazil, etc.) that nonetheless have relatively high-ranked universities. These are usually large and rapidly-growing economies that over recent years have made an attempt to switch from the model of growth based on low-cost labour to a knowledge and innovation-based economy. There are also a number of countries with extremely high GDP per capita levels (Qatar, Kuwait, Luxembourg, and others) that nonetheless have very poor universities. These are usually smaller countries with a lower GDP. Their high levels of GDP per capita are often due to one factor that is not particularly knowledge-intense or innovative, e.g. crude oil exports or a developed financial sector.

These results can also be interpreted in a different way, i.e. from the point of view of methodical specificities of the WUR. Larger countries with greater economic potential would place better in the ranking. In smaller countries even high levels of GDP per capita cannot make up for losses derived from limited resources and a smaller economy. This is one more argument in favour of the limited value of the WUR for the assessment of the quality of the higher education system. U21, which direct measures the quality of higher education, does not have such a defect. The results presented in Tables 2 and 3 indicate that U21 is not dependent on economic potential (in terms of GDP). However, GDP per capita plays a key role. The authors of the ranking also present the data obtained after eliminating the impact of GDP per capita (U21 Ranking 2016).

The results clearly demonstrate that long-term political stability and the continuity of political structures (in other words freedom from war, occupation, coups and major changes in the political system) are among the most prominent factors that determine the position of universities from a given country in international rankings. In contrast to many other organizations, universities benefit from longevity. Much of their knowledge base is tacit, and passed on by masters to their apprentices. For centuries, knowledge was confined to a location (which is why numerous schools of thought were given the name of the city or university, e.g. Wiener Kreis, Chicago School of Economics, Lwowska Szkoła Matematyczna) or suggested a specific location (also through the name of the founder or main researcher). Even now, as new media allow instant and territorially unlimited knowledge transfer and knowledge access, a prominent body of knowledge and skills remains tacit. When universities operate for a sufficiently long time in a relatively stable environment, they are given the opportunity to aggregate suitable social and human capital.

Rapid disturbances to the state structure not only destabilize the academic setting; they sometimes lead to the marginalization or even extermination of scholarly elites, and as such preclude knowledge and skill transfer from one generation to the other. Periods of political instability also upset the fragile balance between the service that universities have to render to society and the autonomy of research from current political processes.

The dummy variable for English-speaking countries proves to be significant in a few of the specifications without the STA variable. This part of the study confirms a cultural rent for English-speaking universities, which is also corroborated by previous research (van Raan et al. 2011). If takes in account STA a coefficient for dummy variable lose statistical significance.

The results reveal the extremely significant role of broadly defined institutional arrangements and governance, especially with regard to public and regulatory policies. The interpretation is limited due to the fact that variables from the World Bank governance data are marked by a relatively high level of mutual correlations, which is why they should be interpreted in total as it represents the institutional environment as a whole. Their significance can be interpreted in two ways. Firstly, universities themselves are subject to regulatory policy and are part of public policy. The poor quality of broadly defined policies of this kind (including the autonomy of the civil service) may produce similar implications for the higher education system. Secondly, broadly defined regulatory and public policy has a primary impact on the quality of the setting of the economic and political environment, thereby having a secondary impact on universities and their international standing.

It is worth pointing out a few limitations of the presented study.

A. The intention was to investigate country-specific determinants of world university rankings. Therefore, the existing rankings, despite all the shortcomings and inherent defects, were subject to the study. The results should, thus, be interpreted cautiously with regards to the actual quality of teaching and research in a given country. The caution is due to the weakness of the rankings themselves. However, the results obtained for all rankings (including those for U21) form a basis for more general conclusions. Nevertheless, it certainly requires further in-depth research.
B. Part of the variables are statistically significant but their impact is relatively weak, as indicated by standardized estimates of parameters. However, it is worth remembering that the study is cross-sectional and thus excluded dynamic effects. Over longer periods of time even variables that have a relatively weak (but significant) influence cannot be ignored, as even small changes cumulated over the years can determine significant differences between countries.
C. The results should not be interpreted in the spirit of (macro) economic determinism. The research was focused on selected macroeconomic and institutional determinants. That said, the results fail to reflect other, e.g. management-related determinants. It is obvious that the higher education system or university governance have an impact on how each university fares in the rankings. However, country-specific factors should also be considered in public debate on university rankings. A rare feature in such a debate, the role of long-term political stability (defined as freedom from war, occupation, coups, and major changes in the political system), is particularly interesting in this aspect.
D. The conclusions for public policy are not clear. Partly, this is due to the purpose and scope of the research itself (which was targeted to indicate country-specific determinants of WUR). Remaining in the scope of the paper, we can say that the policy of public authorities has no effect (especially in the short term and directly) on the part of the variables which determine a university’s place in the rankings, e.g. GDP and political stability. The general public policy conducted in a country is of significant importance—through the quality of institutional arrangements. And this is the first way to improve the international position of universities. The main immediate impact channel (within the test group of variables) involves, however, expenditures on R&D.

## Conclusions

The main goal of the research was to search for country-specific factors that affect world university rankings. We show that the position of universities from a country in the ranking is determined by the following variables: economic potential of the country, research and development expenditure, long-term political stability (freedom from war, occupation, coups and major changes in the political system), and some institutional variables, including government effectiveness and regulatory quality. In other words, there are common factors that explain how universities perform in the WUR. Similar factors (funding, stability and institutional arrangements) affect Ranking of National Higher Education Systems published by Universitas 21. We have also shown some limitations of the presented results.
